# Feasibility and Validity of a Framework for Antimicrobial Stewardship in General Practice: Key Stakeholder Interviews

**DOI:** 10.3390/antibiotics9120900

**Published:** 2020-12-13

**Authors:** Lesley A. Hawes, Jaclyn Bishop, Kirsty Buising, Danielle Mazza

**Affiliations:** 1 Department of General Practice, School of Primary and Allied Health Care, Monash University, Level 1, 270 Ferntree Gully Road, Notting Hill, Victoria 3168, Australia; Danielle.Mazza@monash.edu; 2 National Centre for Antimicrobial Stewardship, The Peter Doherty Institute for Infection and Immunity, University of Melbourne, Level 5, 792 Elizabeth Street, Melbourne, Victoria 3000, Australia; jaclynb@student.unimelb.edu.au (J.B.); Kirsty.Buising@mh.org.au (K.B.); 3 Department of Medicine—Royal Melbourne Hospital, Faculty of Medicine, Dentistry and Health Sciences, The University of Melbourne, Royal Parade, Melbourne, Victoria 3050, Australia; 4 Pharmacy Department, Ballarat Health Services, Drummond Street, Ballarat, Victoria 3350, Australia; 5 Victorian Infectious Diseases Service, Royal Melbourne Hospital, 300 Grattan St, Parkville, Victoria 3050, Australia; 6 Department of Infectious Diseases, Peter Doherty Institute for Infection and Immunity, University of Melbourne, Melbourne, Victoria 3010, Australia

**Keywords:** antimicrobial stewardship, general practice, family practice, antibiotic, health policy, quality of health care, antibiotics, public health, pharmacist, nurse

## Abstract

There is little guidance about developing systems for antimicrobial stewardship (AMS) for general practice. A literature review identified six key components: governance, monitoring of antibiotic prescribing and resistance with feedback to prescribers, consultation support, education of the public and general practitioners, pharmacist and nurse involvement, and research, which were incorporated into a potential framework for the general practice context. Objectives: to determine the feasibility and validity of the proposed AMS framework. A secondary objective was to identify likely bodies responsible for implementation in Australia. We undertook interviews with 12 key stakeholders from government, research, and professional groups. Data were analysed with a thematic approach. The framework was considered valid and feasible. No clear organisation was identified to lead AMS implementation in general practice. The current volume-based antibiotic prescription monitoring system was considered insufficient. AMS education for the public, further development of GP education, and improved consultation support were strongly recommended. The role of community-based pharmacists and nurses is largely unexplored, but their involvement was recommended. A clear leader to drive AMS in general practice is essential for an action framework to gain traction. Monitoring and feedback of antibiotic prescribing require urgent development to include monitoring of prescribing appropriateness and patient outcomes.

## 1. Introduction

Antimicrobial resistance is a global problem with a major impact on health care and associated costs [1]. Exposure of microbes to antimicrobials contributes to the problem [2,3]; unnecessary use of antimicrobials must be minimised. The consumption of antibiotics in the Australian community is high in comparison with similar countries [4], with most antibiotics prescribed by general practitioners [4]. There is a high rate of prescribing of moderate- (66% of use) and broad-spectrum antibiotics (25%) [5], and inappropriate use is still common for conditions such as upper respiratory tract infections [4]. For these conditions, antibiotics are prescribed at rates 4–9 times that recommended by the Australian national antibiotic prescribing guidelines Therapeutic Guidelines—Antibiotic [6]. Australia’s National Antimicrobial Resistance Strategy calls for the introduction of antimicrobial stewardship (AMS) to address inappropriate antibiotic prescribing [7]. However, there is little guidance for how to implement AMS across Australian general practice.

Through a review of international health system approaches to AMS in general practice [8,9,10,11,12,13,14,15,16,17,18,19,20,21,22,23], a potential framework to guide AMS in general practice was formulated. This framework contains six key components: governance, monitoring of antimicrobial resistance and prescribing with feedback to GPs, education for general practitioners (GPs) and the public, consultation support, the involvement of community-based pharmacists and nurses, and research [24]. Details of the framework are provided in Appendix A.

The aim of this study was to interview key stakeholders to determine the likely feasibility and validity of the proposed AMS framework and a secondary aim was to identify any existing organisations who may take on responsibility for implementation in Australia.

## 2. Results

Of the 24 invited stakeholders, 13 accepted. Two declined, another was on extended leave, and eight did not respond to two emails. One of those who declined—despite being invited to participate as an expert, not as a representative—replied, “[name of organisation] is not in the best place to help with your query regarding AMS in general practice and we recommend you contact [another named organisation].” We already had stakeholders from the organisation recommended. One respondent accepted but could not be interviewed in the timeframe. The 12 interviewed stakeholders’ background, relevant expertise, and location are outlined in Table 1. The COREQ checklist is available in Supplementary Table S1.

Overall, stakeholders reported that the proposed AMS framework for general practice and its components were feasible and valid; and that it provided a link between the objectives of Australia’s National AMR Strategy and action. However, most stakeholders highlighted that it would require leadership and prioritisation for implementation to have the desired impact. Importantly, the stakeholders had difficulty nominating the best organisation to oversee this implementation. (Representative quotes are supplied; additional quotes are available in Supplementary Table S2).

It seems very comprehensive to me… able to be implemented… I think we need to have an agreed upon governance structure and agreed upon priorities… I don’t think there is one clear person or group who is responsible for the whole caboodle of this.

(Participant (P) 6)

Asked how they would define success, stakeholders nominated short- and long-term goals. Short-term goals were increased adherence to prescribing guidelines and improved patient outcomes with no increase in harm. Stakeholders also commented that increased professional support provided by such a framework may lead to improved professional satisfaction for GPs. The long-term goals that they stated were a decrease, or at least, no increase in antimicrobial resistance (AMR). 

Governance was reported by the stakeholders to be important to set strategic priorities and harmonize approaches. The importance of aligning work in primary care with work in other sectors was highlighted. A national action plan for AMS in general practice was regarded as a Commonwealth responsibility, with the Office of Health Protection (within the Department of Health) suggested to lead stakeholder engagement.

I think within the implementation plan the Office of Health Protection has an important role… I mean they have the remit of the strategy. In terms of the organisations that will have a responsibility some of them are probably clear, and some of them just need coordination. The important part of that is to work in a collaborative way, coordinated way… We shouldn’t be… isolating sectors such as hospital, aged care… primary care.

(P5)

There were calls to make practice accreditation mandatory and to include AMS activities such as antibiotic monitoring or education in this. Suggestions were made for financial incentives to encourage AMS activity in general practice.

Stakeholders also generally supported greater regulatory controls on prescriptions, the removal of automatic repeats, and promotion of unit dispensing (dispensed quantities match antibiotic guideline recommendations, not pack sizes).

People you can educate as much as you like, but until you actually restrict the antibiotics people aren’t going to stop using them…

(P6)

Monitoring and feedback on antibiotic prescribing was perceived as effective for changing behaviour, but the current process was viewed as problematic. Unresolved practical considerations included that complete datasets are not available, the possible defensiveness of GPs about their data being reviewed, questions about who would analyse and provide feedback to GPs, and whether collection should be mandatory or incentivised. The government was regarded as responsible for obtaining complete datasets. Stakeholders saw potential for the Practice Incentives Program—Quality Improvement Incentive [25] (GP data collected by the Primary Health Networks (PHNs) for process measures) to include antibiotic monitoring. Stakeholders said that feedback should include peer comparisons, and ideally link in with education and consultation support. The potential use of positive variance was described, that is, investigating the strategies used by those who prescribe fewer antibiotics than their peers.

Government needs to incentivize, to capture [antibiotic prescribing] information. You know organisations like the PHNs are really well suited to that.

(P11)

In terms of investigating what works, one thing that we do poorly is to look for positive variance.

(P7)

Community education in the form of ongoing tailored public health campaigns was considered important and viewed as a government responsibility. There were suggestions that health literacy education for antibiotic awareness should start at school.

We do need the consumer to come on board to… not have that expectation [for antibiotics], which then does make the consultation very difficult.

(P8)

GP education endorsed by the Royal Australian College of General Practitioners (RACGP) or supplied by PHNs or medical specialists was well regarded and trusted. NPS MedicineWise (an independent organisation supporting quality use of medicines) was acknowledged as an existing channel for GP education, but it was questioned as to whether what was currently provided was at the depth necessary to have the largest impact. There were concerns that pharmaceutical marketing may undermine AMS messages.

What type of education do GPs trust? And often that’ll be one that comes from kind of RACGP-branded things, or PHNs, and sometimes specialist.

(P4)

Stakeholders wanted improved clinical software that integrated prescribing guidelines, patient information resources, and alerts. There was a suggestion that some GPs are using product information rather than guidelines to inform decision making because unlike guidelines, product information is integrated into the clinical software. Government-funded health services (e.g., NPS MedicineWise, PHNs) were suggested as potential developers of patient information resources with PHN Health Pathways as another potential host to make the resources widely available. Keeping the resources current was identified as a challenge.

I think electronic decision support can work well if it’s in real time…. the first line choices of antibiotics are… if you couple that with patient information that will be… made available to the patient, that’s helpful.

(P7)

Rapid and point-of-care tests elicited mixed comments. Some thought these could be useful if subsidised. Others thought they should only be available if it would change the decision to prescribe an antibiotic. Selective reporting of antibiotic susceptibilities was suggested as a priority along with standardised information for GPs about the use of microbiology testing, particularly around specimen collection and interpretation of results. It was suggested that the Royal College of Pathologists of Australasia (RCPA) should oversee this.

Not all labs do selective reporting of antibiotics; it should be implemented… we need one official form rather than lots of different ones—they are not as strong as one consistent message.

(P12)

Expert advice sought from hospital specialists (including infectious diseases consultants) was often based on relationships developed during training. There were calls for a central advice line, or lines of communication to enable consistent messages or access to the local hospital specialist’s guidance.

Expert advice for me is very dependent on relationships that I built when I was in the hospital system. So if you’ve got a good network of experts you can call on but you know from an infection perspective it’s… reliant on the goodness of… them giving you their time…

(P6)

…whether or not the government would be interested in having access lines for antibiotic resistance… if someone could ring them up … and get advice, probably wouldn’t be a bad thing.

(P8)

Respondents suggested that adding the reason-for-prescription (subject to privacy requirements) and providing an exact duration of antibiotic therapy to the prescription would help community pharmacists be more engaged in AMS. It was perceived that to successfully implement delayed prescriptions (where the patient is told when and under what conditions antibiotics should be dispensed), better communication between GPs and community pharmacists is needed. Pharmacists employed by the general practice were identified as an opportunity for practice-level AMS support.

[pharmacists] *put a sticker on the box of antibiotics that says finish the course… we should change the stickers to ‘take as long as prescribed’…*

(P8)

Allergy testing was regarded as beneficial for individual patients but not a system issue. Handover of antimicrobial prescribing on patient transfer was considered part of the larger issue of handover of all information. 

Stakeholders thought that nurses may have a role in AMS, e.g., patient triage and education in the community and in the practice, but there was a perceived lack of funding.

I think [nurse triage is] fantastic in an ideal world, but we don’t have the funding.

(P8)

Stakeholders agreed that research into general practice AMS with translation of the evidence into practice was required. Research areas suggested included understanding the potentially negative effects of antibiotics on the gut microbiome, and better understanding of the use of delayed antibiotic prescription “*whether an illness that’s been present for more days is more likely to respond to antibiotics*” (P7). Stakeholders also suggested more research to understand low prescribing GPs:

Those who seem to manage to preserve this resource [antibiotics] really well and apparently not with any problems in terms of the health of their patients. Yeah. How does it work for them? What helps them, what supports them? What can we put in place to enable others to not prescribe?

(P9)

## 3. Discussion

Stakeholders agreed that the proposed framework was valid and feasible, and provided a suitable action framework for the introduction of AMS into Australian general practice. Central coordination was identified as a priority, but the lack of clarity around who would provide this leadership was surprising, particularly given the seniority of the participating stakeholders. The Office of Health Protection (OHP) was suggested to lead and coordinate the introduction of AMS into Australian general practice. Whether the OHP has the capacity for this was not investigated. Sweden’s Strama program offers an example of leadership at county and national levels [17,18,26].

Monitoring of and feedback on antibiotic prescribing will enable targeting and evaluation of AMS interventions. Several issues were highlighted including GP trust in a transparent external audit process [27] and a need to obtain complete datasets (including the reason-for-prescription in a standardised format). Inclusion of information on any adverse patient outcomes, e.g., hospitalisations, would require linkage of datasets [27]. There was a view that monitoring and feedback needs urgent development beyond the current volume-based feedback so that it better meets clinical need. No current monitoring system was identified that could provide the information required. An example of monitoring and reporting are the annual reports published by the English Surveillance Programme for Antimicrobial Utilisation and Resistance (ESPAUR) [28].

Regulatory changes were supported. Manufacturer’s pack sizes rarely match the recommended duration for common conditions [29], and when antibiotics were supplied by the pack, patients were thought to be likely to save leftovers for future use [30]. Restrictions on repeat prescriptions for five of the most commonly prescribed antibiotics in Australia were introduced on 1 April 2020 [31], illustrating that regulatory changes are achievable.

Electronic decision support was strongly supported and should be further examined in Australia. It has been used to guide prescribing in hospitals, and has been effective at reducing antibiotic prescribing when combined with other AMS interventions [32]. Work is required to develop and pilot suitable electronic decision support to ensure that the tools meet prescriber needs in Australian primary care, are usable, fit in with workflow [33], and have the desired impact. 

Stakeholders were unanimous that community education is required to support general practice AMS. Evidence suggests that campaigns may work best when developed in partnership with consumer organisations, are coordinated with health professionals, and promoted at local and national levels [34]. Community awareness of a common colds campaign reflected changes in the frequency of the campaign [35], suggesting that community education should be ongoing. School-based programs, such as Europe’s eBug [36] and Canada’s Do Bugs need Drugs? [37], have introduced AMS to children. Alongside community education, the provision of written patient information was widely supported by stakeholders and has been associated with reduced antibiotic prescribing in common infections [38]. However, the issues of updating the information, which languages and cultural information are required, and the most appropriate place to host these have not yet been well addressed in the literature. 

Ongoing work on selective reporting of antibiotic susceptibilities by microbiology services, which has been shown to be effective in influencing prescribing behaviour [39], should be pursued as a priority in Australia [40].

Increased access to expert advice has been utilized internationally as a method to influence antibiotic prescribing choices. Telephone advice has been provided to GPs in France for patient management [22] and in Sweden, experts provide advice on interpretation of audit results [18]. While stakeholders supported the provision of centralised expert advice, there was no clarity on who should provide it beyond the suggestion that local hospital specialists might participate. 

Internationally, pharmacists have participated effectively in activities to help reduce antibiotic prescribing and increase prescribing guideline concordance [41], but Australian community pharmacists may require additional support for this expanded role [42]. Non-dispensing pharmacists in general practice may be suitable for an AMS role. Research to explore the role of pharmacists in general practice AMS is recommended. The role in AMS of practice nurses and that of nurses in the community (e.g., phone triage lines) and their need for formal AMS education remains largely unexplored.

Allergy testing and handover of antimicrobial prescribing on patient transfer will be removed from the framework as the former is an individual issue and the latter part a broader issue. No other changes were recommended.

There are limitations to this research: the recruited practice nurse stakeholder was unavailable for interview in the timeframe, so there may be additional insights to be gained regarding the involvement of practice-based nurses. There were only 12 interviews conducted and stakeholder identification was partly reliant on the authors’ networks. Areas covered in less detail were the roles for specific organisations in implementation. The RCPA and the Office of Health Protection were specifically named by one stakeholder for each. However, other stakeholders referred more generally to the “professional colleges” and “Department of Health”, respectively. Components in which only three stakeholders commented were: planning for new antibiotics, the role of allergy testing, handover of patient information, unit prescribing, and knowledge about other AMS models. Components discussed by four stakeholders included: pharmaceutical company marketing, nurse involvement, monitoring of AMR. All other components were discussed with at least five stakeholders.

The views of the expert stakeholders may not reflect those of the wider GP community. Experts are likely to be early adopters or innovators in a field [43], whereas the wider community will include those who fear the consequences of not having antibiotics and those who may not perceive that AMR affects them. The stakeholders were speaking as experts, not as representatives of their organisations, thus it is unknown if the organisations have the current capacity to implement the framework.

I should just say… I’m not doing this from [a named organisation] policy view.

(P3)

This is a health system-wide framework developed from a review of the international literature [24] which identified components that may play an interdependent role affecting GP antimicrobial prescribing. A systematic review of interventions found that “No single intervention can be recommended for all behaviours in any setting” and that “local barriers should be removed before implementation” [44]. Examination of these components may help to explain why an intervention may be successful in one setting but fail in another [44,45]. This research highlights that AMS in general practice needs a health system leader, the involvement of health departments, especially One Health AMS committees, with input from professional colleges and health professional representatives. Implementation science and behaviour change principles [46,47,48] with GP and relevant professional input are recommended to pilot, implement, and evaluate changes.

## 4. Materials and Methods 

### 4.1. Study Design and Participants

A qualitative approach was used. Australian-based senior expert stakeholders in AMS in general practice were identified through the authors’ AMS networks (8), relevant organisations’ websites (3), and via contact with government and professional organisations (2). Stakeholders were provided with a study information sheet and purposively invited to participate in a telephone interview. Gift cards to the value of AUD 150 were offered as compensation for their time. 

### 4.2. Data Collection and Qualitative Analysis

Consented participants received an outline of the proposed AMS framework prior to the interview (Appendix A).

In-depth telephone interviews using a semi-structured interview guide (Appendix B) were conducted and recorded between September and December 2019. Stakeholders were purposively invited until key components had been discussed with at least one stakeholder. Feasibility and validity were assessed by asking participants the extent to which components and subcomponents were being done or if plausible, what needed to be done to make them implementable; their priorities; and if they could identify any gaps. Data collection was completed before analysis commenced. Interview recordings were transcribed and returned to stakeholders with a 10–14-day window for amendments. Transcripts underwent thematic analysis using deductive coding targeting comments about the proposed framework and its components, and by open coding for other comments [49]. Two transcripts were independently coded by two authors and an agreed coding framework was developed. Three more interviews were dual coded using the agreed framework and adjustments made. Seven transcripts were coded by one author. NVivo 12 qualitative data analysis software (QSR International Pty Ltd. Chadstone, Australia) was used to manage the transcripts and coding. 

Ethics approval was granted by the Monash University Human Research Ethics Committee, number 20721. 

## 5. Conclusions

The stakeholders regarded this AMS framework as feasible and valid for Australian general practice. The individual subcomponents were viewed as providing a link between the objectives of Australia’s National AMR Strategy and action. However, stakeholders considered that the framework required an implementation process with priorities and an integrated approach. The identification of a clear leader to drive AMS in general practice is essential for AMS to gain traction. Monitoring and feedback of antibiotic prescribing require urgent development beyond the current volume-based system and should include monitoring of appropriateness of the prescriptions and patient outcomes. AMS education for the public, further development of GP education, and improved consultation support were strongly recommended. The role of community-based pharmacists and nurses is largely unexplored but their involvement, particularly for patient education, was recommended. Several areas for research were suggested.

## Figures and Tables

**Table 1:** The professional background, antimicrobial stewardship (AMS) involvement, and location of the 12 interviewed stakeholders.

Professional Background (Not Necessarily Current Employment)	Number
General practitioner	6
Pharmacist	5
Medical Microbiologist	1
**TOTAL**	**12**
**AMS Involvement** (Stakeholders may have multiple roles)	
Clinical Quality Improvement/AMS committee/professional organisation representative	9
Researcher in general practice AMS	4
Health Department (including Public Health)	2
Primary Health Network	2
Microbiology Laboratory	1
**Location**	
New South Wales and/or Australian Capital Territory	4
Victoria	4
Queensland	3
Tasmania	1
**TOTAL**	**12**

## Supplementary Materials

The following are available online at https://www.mdpi.com/2079-6382/9/12/900/s1, Table S1: The COREQ checklist, Table S2 Representative quotes for AMS components.

## Appendix A. Component List Used during the Interviews

The detailed list of the subcomponents for antimicrobial stewardship in general practice. This list was sent to each Stakeholder before interview and referred to during the interview.

1. Governance
  - National action plan;
  - Antimicrobial resistance included on national risk register;
  - Multi-level and/or multi-disciplinary response;
  - Regulations around antimicrobial stewardship and antibiotic prescribing;
  - Accreditation of prescribers;
  - Funding for antimicrobial resistance and stewardship activities;
  - Planning for release of new antibiotics;
  - Practice level antimicrobial stewardship policy/program/activities;
  - Handover of antibiotic information.
2. Education
  - Community and patient education;
  - GP continuing education in antimicrobial stewardship;
  - GP education on communication skills, patient-centred approaches and shared decision making;
  - GP education on non-antibiotic management of self-limiting infection;
  - GP education on delayed prescribing;
  - General practice team member education;
  - Independent education (restrict pharma marketing).
3. Consultation support
  - Prescribing guidelines;
  - Point of care tests;
  - Microbiology testing and reporting;
  - Allergy testing;
  - Electronic decision support for prescribers;
  - Expert advice;
  - Decision support for use with patients.
4. Allied health support for antimicrobial stewardship
  - Unit dispensing;
  - Supply and timely access to antibiotics;
  - Pharmacy review and advice;
  - Appropriate disposal of leftover antibiotics;
  - Nurse triage, patient assessment and education.
5. Data monitoring
  - Monitoring of antibiotic prescriptions;
  - Monitoring of antimicrobial resistance;
  - Feedback to prescribers and reporting.
6. Research
  - Research into AMR/AMS gaps, translation into practice.

## Appendix B. The Semi-Structured Interview Guide

1. What can you tell me about your interest or experience in antimicrobial stewardship?
2. What do you think is required to improve antibiotic prescribing in general practice?

Now I will take 2–3 min to explain the model framework and then I will ask you for your comments on it.

3. What is your overall impression of this framework?
4. How well does each component reflect what you understand about AMS?
5. Is it plausible?
6. Does anything not ring true?
7. Do you know of any other models?
  - How do they differ from this model?
8. To what extent are each of these components currently being done?
9. To what extent do you think the other components are implementable?
  - What needs to be done to make it happen?
10. Who is, or should be, responsible for each of these components?
11. What do you think may happen if all this came to be?
12. Are there any gaps in this framework?
13. What would you prioritise?
14. How do we measure success? (Interviews 6–12 only)
15. Is there anything missing that we haven’t discussed?
