# Supplementary material for: Feasibility and Validity of a Framework for Antimicrobial Stewardship in General Practice: Key Stakeholder Interviews
Source: Antibiotics (Basel). 2020 Dec 13;9(12):900. doi: 10.3390/antibiotics9120900 (PMC7764540; doi:10.3390/antibiotics9120900)
Supplement: Supplementary file 1 [file antibiotics-09-00900-s001.pdf]

antibiotics

MDPI

Supplementary Materials

Article

Feasibility and Validity of a Framework for Antimicrobial Stewardship in General Practice: Key Stakeholder Interviews

Lesley A. Hawes [1,2,*], Jaclyn Bishop [2,3,4], Kirsty Buising [2,5,6] and Danielle Mazza [1,2]

1 Department of General Practice, School of Primary and Allied Health Care, Monash University, Level 1, 270 Ferntree Gully Road, Notting Hill, Victoria 3168, Australia; Danielle.Mazza@monash.edu
2 National Centre for Antimicrobial Stewardship, The Peter Doherty Institute for Infection and Immunity, University of Melbourne. Level 5, 792 Elizabeth Street Melbourne, Victoria 3000, Australia; jaclynb@student.unimelb.edu.au (J.B.); Kirsty.Buising@mh.org.au (K.B.)
3 Department of Medicine—Royal Melbourne Hospital, Faculty of Medicine, Dentistry and Health Sciences, The University of Melbourne, Royal Parade, Melbourne, Victoria 3050, Australia
4 Pharmacy Department, Ballarat Health Services, Drummond Street, Ballarat, Victoria 3350, Australia
5 Victorian Infectious Diseases Service, Royal Melbourne Hospital, 300 Grattan St, Parkville Victoria 3050, Australia
6 Department of Infectious Diseases, Peter Doherty Institute for Infection and Immunity, University of Melbourne
* Correspondence: Lesley.Hawes@monash.edu

Table 1. The COREQ checklist.

	**Item**	**Response**	**Section reported in**
1	Interviewer	LH conducted the interviews	Methods
2	Credentials	LH: BSc(Hons), MPH, PhD candidate<br>JB: BPharm(Hons), MPH, PhD candidate<br>KB: MBBS MD MPH FRACP<br>DM: MD, MBBS, FRACGP, DRANZCOG, Grad Dip Women's Health, GAICD[1]	
3	Occupation	LH: Microbiologist/PhD fellow<br>JB: Pharmacist/PhD fellow<br>KB: Infectious diseases physician/Researcher<br>DM: General Practitioner/Researcher	
4	Gender	Not relevant to this study	Not Applicable
5	Experience and training	LH and JB have undertaken training in qualitative research and have previous experience with qualitative research.<br>KB and DM have conducted, supervised and published qualitative research.	Available on request
6	Relationship established	LH/KB/DM identified potential stakeholders. LH conducted recruitment by formal invitations	Methods
7	Participant knowledge of the interviewer	Some stakeholders had previous professional contact with one or more of the research team.	Methods

8	Interviewer characteristics	The invitation to participate explained the purpose of the study, and who and what was involved.	Methods
9	Methodological orientation and theory	Qualitative research	Methods
10	Sampling	Purposive sampling through professional networks.	Methods
11	Method of approach	One email containing explanatory letter and consent form; non-responders were sent one repeat email. Written consent was obtained for the interview.	Methods
12	Sample size	13 participants	Results
13	Non-participation	One participant could not be interviewed in the timeframe.<br>One stakeholder refused to participate.	Results
14	Setting of data collection	Telephone interviews	Methods
15	Presence of non-participants	The interviewer was alone in a private office. Interviewees chose their own setting.	Not applicable
16	Description of sample	Background profession, State, and experience in AMS	Results
17	Interview guide	A semi-structured interview guide was pilot tested on 3 volunteer GPs, adjusted and one extra question was added after interview 6.	Methods and Appendices A and B
18	Repeat interviews	No repeat interviews were conducted	Not applicable
19	Audio/visual recording	One participant did not consent to recording. Permission was obtained to make notes during the interview. They were transcribed immediately after the interview. All other interviews were recorded.	Methods and Results
20	Field notes	Field notes were made during and after the interviews	Available on request
21	Duration	Interviews lasted between 39 and 68 minutes	Results
22	Data saturation	All components were discussed; those in less detail were specifically named bodies, and the risk register.	Discussion
23	Transcripts returned	Transcripts were returned to all participants	Methods
24	Number of data coders	2 coders: LH and JB	Methods
25	Description of the coding tree	Coding was in two parts:<br>Deductive codes derived from the proposed framework.<br>Open coding for other themes - adding inductive codes to the coding tree.	Methods
26	Derivation of themes	Thematic analysis; deductive and open coding	Methods
27	Software	NVivo 12	Methods
28	Participant checking	All transcripts were returned to participants with a 10-14-day window for amendments or withdrawal.	Methods
29	Quotations presented	Yes	Results and S2.
30	Data and findings consistent	Yes	Results and Discussion

31	Clarity of major themes	Yes	Results and Discussion
32	Clarity of minor themes	Yes	Results and Discussion

antibiotics MDPI

Table 2. Representative quotes for AMS components.

**Perceptions of the model framework**
It sounds comprehensive… there wasn't anything that stood out to me as a significant gap or a gap… I guess the part of the model and I'm sure you're considering it's how you actually support GPs in implementing a lot of these things that you're sort of outlining. (P5)
I think it sets a very nice summary of all the angles and approaches that have been tested and trialled…I think it's a really nice model. Well done. (P9)
I think it's a useful way of kind of divvying up the different elements that need to happen. (P3)
**Governance: National Strategy and Action/Implementation Plan**
**Leader, input**: National Action Plan is generally led by the Commonwealth [Department of Health] but requires that input from all of the other stakeholders including jurisdictions and GPs and everyone else. (P3)
**AMS across sectors**: Many GPs also service aged care facilities as well… In the more rural remote areas, the GPs there work both in the hospital setting, the community and the aged care setting... GPs in those areas [are] happy to do antimicrobial stewardship, but... don't want to have a different model for every place that we're working in; we want to be saying the same message in the same strategy in the same model. (P10)
**Unclear responsibility**: So we've got the National Action Plan [National AMR Strategy] which I suppose is the agreed upon priorities for the country… But also knowing that there's no way that one agency can fix this…. Everybody's problem and nobody's responsibility. I don't think there is one clear person or group who is responsible for the whole caboodle of this. (P6)
**Implementation**: We need some kind of a national action plan. But you know a lot of these action plans are mostly pieces of paper if it doesn't trickle down to something on the ground and so I think that that is an important part. But you need the middle layer, the regional health services and PHNs in the sense of primary care. They all need to be on board and have a panel report and then all the way down to the practice level where GPs operate… But each action, each policy needs to have you know a number of elements that show how it is being implemented, and how it is being monitored, if that implementation is happening or not. So, you need a full circle process... we're first of all on the same page, everyone knows about this, everyone knows their responsibilities, that there is a clear action plan on how we can make this all happen, and that we are then accountable for how that happens at each of those. (P9)
**RACGP role**: I think [RACGP] have a role in looking at what other policies are recommended by external people including Department of Health and seeing how it would play out in the different environments that GPs work in. From a rural remote Aboriginal or Torres Strait Islander Health, tropical health, urban health. So it's a…set of eyes on policy decisions. (P7)
**Professional College involvement in messages**: it's got to be global and then come down through… your different organizations that you belong to (P1)
**Tailoring of messages**: It's not about controlling GPs, it's about better outcomes for your patients and better outcomes for our antibiotics in the longer term. (P2)
**Governance: Regulations**
**PBS**: How the PBS matches recommendations. Whether you can actually access the correct antibiotic for the correct length of time according to recommendations under the PBS. (P3)
**Repeats**: We need to stop putting repeats on things like prescriptions, I mean that's a no brainer. That's just going to happen. (P6)
**Off label prescribing**: looking at tightening up the regulation around… prescribing things off label. I think is a big problem and you know potentially should just not be allowed. (P6)
**Expiry on antibiotic prescriptions**: we have a 12-month expiry on prescriptions and that would be a big legislative change to change antibiotics from that 12-month expiry. So I think if we could have some sort of clause in the antibiotic prescribing say if this prescription has not been presented within two weeks you know do not fill. (P1)
**Authority to prescribe antibiotics**: but what missing piece was you know the regulations; the governance and I think they're very… powerful drivers. For instance, if we look at our quinolone prescribing in Australia are very very good, and so not prescribing lots of quinolones in primary care, so our resistance patterns are very very good. And why is that? Well because you need authority to prescribe a quinolone. And that's a barrier. Oh, it's the regulation. Having those barriers work (P9)
**Governance: National risk register**
[AMR has] become a Tier 1 priority on… the Australian Health Minister's Advisory Committee… I believe. (P6)

**Governance: Accreditation of prescribers and/or general practice**
**Prescriber accreditation for evidence-based practice**: I think the years of doctors being completely autonomous and just doing whatever the hell they like are gone… There needs to be a degree of responsibility for undertaking evidence-based practice. And perhaps even consequences for not doing so… Stuff like do maybe… a stewardship module in RACGP or ACRRM CPD programs and do you make it mandatory? You know if you want to prescribe X, Y, Z antibiotics you have to have done this module. (P6)
**Prescriber accreditation for monitoring or education**: If you're a GP prescriber of antibiotics you probably do need to be you know compulsorily reviewing or auditing or at least attending some sort of updates about antibiotic prescribing every three years… I would really like to say that is a compulsory part of CPD. (P2)
**Mandatory practice accreditation**: Accreditation definitely needs to play a role. I know they have got a voluntary accreditation process, but it should be mandatory (P10)
**Governance: Funding**
**Cost of not funding AMS**: I actually think that health economists need to look at the consequences of not funding antimicrobial stewardship - so that the potential that an elective surgery and immunosuppression become too dangerous to contemplate. And the cost of untreatable infections and managing those people in isolation. (P7)
**Public funds:** There's a decent economic argument to a lot of the actions that they passed on climate change. But [AMS] is never going to be an exercise which makes money. So there's always going to have to be a degree of public funds involved in it. Because antibiotics are the epitome of the market failure aren't they?... But I suppose primarily government and the regulating bodies have got the overarching responsibility and when the s**t hits the fan in a big way it will become their problem. (P6)
**MBS funding** I was thinking at a very high level that MBS funding changes so that funding isn't necessarily just tied to the time of the consultation because obviously you know what the main issue is the fact that it's faster to write out a prescription then go through that communication process explaining why the person doesn't need antibiotics. (P4)
**Funding for AMS activities**: I think we need to be realistic, that I suspect it's unlikely that anybody is going to turn around and say we've set aside five hundred million dollars to fix AMR in this country. I think we need to have an agreed upon governance structure and a grand agreed upon priorities and then a bunch of things that are ready to go if and when smaller chunks of funding become available. (P6)
**Incentives**: I guess how practices implement… you can somehow sort of incentivize a level of minimum Implementation or standardization or mandate some of that. (P5)
**Governance: Planning for release of new antibiotics**
if you're a GP prescriber of antibiotics you probably do need to be … compulsorily … auditing or at least attending some sort of updates about antibiotic prescribing every three years … where they can give stuff about appropriate choice of antibiotics and give information on perhaps the new antibiotics when they are appropriate or not appropriate so. I would really like to say that is a compulsory part of CPD. (P2)
**Governance: Practice level antimicrobial stewardship policy/program/activities**
**Practice level policy development**: At a practice level the antimicrobial stewardship policy I think that's underdeveloped. (P4)
**Implementation support**: How you actually support GPs in implementing a lot of these things that you're sort of outlining… to make it easy for them to do that and part of that I think is like how do you bring people within a practice together to agree on how they're going to do things. (P5)
**Whole of practice approach:** I'm just wondering if those discussions [monitoring and feedback of prescribing] you know really should be held at the practice level NOT at the GP in the practice… because antimicrobial stewardship is part of the whole practice and should be owned by everyone in the practice. So you know this could be a nice model of looking at how we provide quality care by focusing on the team and all the different members of the team rather than only the prescribing ones…. (P9)
**Governance: Handover of antibiotic information**
**Handover between health facilities**: Handover antibiotic information, kind of belongs with the hospitals in terms of that handover thing, but it's clearly a much bigger issue than just antibiotic information, so it will get caught up in bigger, bigger communication things around that. And if just done as a antimicrobial stewardship thing probably isn't going to be terribly effective cos it's got to capture the bigger handover issue. (P3)

Education: Community and patient
**GPs and patients together**: Alongside any attempt to shift the dial with GPs, you need to shift the dial with patients as well, so that patients aren't wanting antibiotic and GPs not wanting to prescribe them, and then you see a drop off because they can both be on the same side. (P7)
**Responsibility for and evaluation of campaigns**: Having an idea of what the community understands about resistance and infections and antibiotics and prescribing and then you know monitoring that as education campaigns and that kind of things go up… the only ones that I'm aware of are the ones that have been done by NPS MedicineWise… And there's a website, a federal website… that's one of the sticking points, is it a state responsibility or a federal responsibility and I think there's been a lot of hand balling and somebody just needs to pluck up and say we'll do it… health promotion groups who lead these kinds of campaigns you know so in my head it's a public health campaign and I feel like some of the money that's traditionally gone to non-communicable diseases could potentially go to some of this kind of thing. So yeah anybody really, as long as it's evidence based and the appropriate stakeholders being consulted to make sure there's no unforeseen adverse outcomes. (P6)
**Relevant message**: reaching consumers at a time that's relevant to them through mediums that they use requires multiple channels to be used, multiple you know sort of messaging, messaging appropriate to that consumer. And so it needs quite significant funding… and sustained over a period of time and working with communities at community level is important. So You know what's relevant to students as they going through exams versus what's relevant to a retiree… you just can't have the same messages. (P5)
**Responsibility for messages**: I think the National Prescribing Service; I think we should almost have you know the government messages as well like… government ads regarding obesity over the years. I don't think this is any different. Now we have government messages about don't go to the emergency department. You know I don't see why we're not having government messages… I don't think that's a difficult campaign for the government do. And I think they really need to probably step up and do it... I think it's important that community organizations like the Consumers Health Forum [are involved]. (P8)
**Timing of messages**: [Antibiotic Awareness Week] ties in with the Northern Hemisphere. And I think we should move that to April… and talking about getting your vaccination for flu. It's a virus. Often it does not need to be treated by antibiotics… I just think there's got to be more education around March-April. So whether it ties in with the vaccination campaign and talks about antibiotic resistance and you know prevention is better than cure and that sort of thing... I think it should be twice a year and regularly twice a year. (P1)
**Ongoing campaigns**: we do campaigns but they're not ongoing. And so you might sort of educate one group of consumers but then the next group of young parents comes along and they maybe aren't so informed and so I think it just needs to be continuous never ending campaign of awareness for consumers. (P8)
**In schools**: Some of the aids that are available can still be quite confusing for patients with poor health literacy. And that's where actually that goes back to education, that perhaps more needs to be done in schools and even kinders [kindergarten, pre-school] regarding antibiotic use. (P4)
**Personalised messages**: I think the conversation needs to change to a personal pros and cons. So how long will you and your family be exposed to resistant organisms as a consequence of this antibiotic? What are the pros and cons for your patients as an individual, on having antibiotics? What's the natural course of the disease they've got without? What are the effective safety netting erm discussions to have? And we know from some really good research that the GPs have a belief that antibiotics are much more effective than they actually are. Patients have the belief that's even more far from the truth. And so attacking the beliefs and skills probably makes more difference than tackling the pure knowledge. (P7)
**Consistent messages**: I think you know the government has a responsibility for it… But I think it all should be coming from the same sort of platform that everyone should have the same message. But maybe with organizations in their actual affiliation should be delivering them… like the ASTAG AMR… Well whether it's… the NPS that starts it. And they're given you know resources to be able to do that. And then they can deliver it down to doctors, pharmacists, nurses, and community. (P1)
**GP continuing education**
**Good for our patients**: I guess it's really a matter of getting the right people to do [education] number one; but secondly not being seen as if it's something that is being done as an imposition on GPs. It's something that's being done because it's really good for our patients and good for the future of our prescribing tools, antibiotics. (P2)
**Assumed patient knowledge**: I wonder if people who've been doing it for longer and you know much more experienced perhaps forget that patients don't necessarily know these basics [about non-antibiotic management of self-limiting infections]. We assume, sometimes I suspect we assume, knowledge that is not necessarily there. (P6)
**Communication re self-limiting infections**: Working out the best way to deliver that management, non-antibiotic management, of the self-limiting infection is probably a really key thing that I reckon that we could work on - that communication, the best way to communicate that message… I guess shared decision making… also making more awareness of the increasing number of bugs that… can be self-limiting. (P2)

antibiotics

MDPI

**Easy and customised**: I guess you've got to look at making it as easy as possible for [GPs] to participate… I think there's a need for some national coordinated effort to see national objectives being met but there's also a need for being up to provide educational opportunities which may be more variable to different needs, different level, different regions. (P5)
**Integrated with resources**: I think it works best if it is an integrated approach. So to me there's no point in providing more education if decision aids aren't available or if unit dispensing doesn't change. (P4)
**Education vs behaviour change**: the standard approach to any wicked medical problem is to is to say let's educate GPs more. It doesn't have a long shelf life once you've educated GPs because you need to ask the question why is the prescribing happening? And then tackle the why's... But I'm not sure there's a massive lack of knowledge, but there might be a belief system that needs to change. (P7)
**Education around beliefs**: As part of your education you would explore those backgrounds and beliefs… that they have developed throughout their training and how that aligns with what we're trying to achieve here. So I think it starts with that conversation in making those underlying beliefs and attitudes visible. And once you have named them and brought them out in the open that's when you can start addressing them. If they stay implicit and under the surface whatever you do is going to be cosmetic. (P9)
**Depth and impact**: NPS does education… But are they doing it in the depth that needs to be done… and impact as well you know, do some of these give bigger bang for the buck than others? (P11)
**Microbiology**: GPs need education with how to use tests, which to do, which are urgent and communication with the lab. There is not much literature on what GPs need or what supports they need. Some tools are more powerful – GPs should be told. (P12)
**AMS education for practice team members and allied health**
**Providers and nurses**: In terms of who should do it… I think you know profession-led is the way … I think in terms of everyone being on the same page… When the flu season comes around every year the Public Health Unit will put on the education for the nurses and GPs because you know it's part of the vaccination program. (P8)
**Joint education**: Many PHNs have an education program that includes both pharmacists and GPs.… what's been quite successful is… where you have a joint education… workshop so making it more interactive… and getting the pharmacists involved in a whole practice approach to how the patients are going to be managed within the GP practice, because obviously you want the same message going out and the GP saying one thing, you want the pharmacist to support it and vice versa. (P10)
**Whole of practice education**: General practice team member education I think is lacking there. I doubt that many receptionists would have received training about antimicrobial resistance. So I suppose that could go back to linking antimicrobial stewardship to accreditation, as accreditation activities include the whole practice. (P4)
**Nurse-patient communication**: if [nurses] see something that they think needs antibiotics, not saying that but saying I'll just get the doctor to have a [look]. (P8)
**Independent education (restrict pharmaceutical company marketing)**
Yeah we're pretty good at not advertising direct patients. There are subtle ways it goes on still. You know GP offices are littered with trade names of antibiotics in general. (P7)
The ads that are in GP magazines. … I do think that sometimes they put inappropriate [advertisements] like Fosfomycin was really being pushed as a UTI treatment and it's not really appropriate to apply… making it clear if they recommending stuff that's not in the antibiotic guidelines for the type thing then that would be really useful to have it as a footnote on that. … this antibiotic is not on the PBS is publicized, but they don't say this antibiotic is not recommended first line by antibiotic guidelines. (P2)
**Consultation support: Electronic decision support for prescribers**
**Automated recommendations**: We've just got to get the right tools and the right prompts in place you know so a doctor makes a diagnosis, why doesn't Therapeutic guidelines pop up with the right clinical resources around prescribing for example, the right duration. (P11)
**Reason-for-prescription**: I think the issue is that medical records served serve two purposes for the GP. One's an aide memoir for the next consult. You know, a little summary of what's happened, and the other's a sort of medical legal record in case something goes horribly wrong. And given that that's the function of them why would anyone put time and effort into completing fields that aren't part of that, those two requirements? (P7)
**Software improvements**: software companies generally only change from feedback from GPs. So they need to come as a groundswell of… of GPs using the software to… do their education and go that and think oh well we'd like to have reporting on how we're doing, but we… notice that we don't put reasons-for-prescription in because of these reasons and then they write to the software prescribers to get that changed. (P3)

antibiotics

MDPI

**Consultation support: Prescribing guidelines**
I still think a lot of GPs may use the product information [PI] for their information on prescribing because it's integrating with your GP software... And as you may know that the evidence that's informed what's in the PI is often way out date particularly if you look at penicillin and stuff like that, they've obviously been registered under the TGA for potentially decades and the information the PI is usually related to that the initial registrations so often the indications that are wildly out of date or been superseded and certainly the dosing when it comes to children but even often even in adults has being superseded by increased evidence... So I do think that's somewhere where getting something like Therapeutic Guidelines or some similar evidence based up to date evidence based software integrated into the GP prescribing software would be a key thing that we could do so that you know based on the GP that choosing the best antibiotics and using the right dose and using the right duration because PI doesn't actually have much of that duration that type thing. (a GP)
**Behaviour change:** TG has also put out you know a good summary of antibiotics prescribing. The National Prescribing Service also has that small resource, so I think the resources are there. I think it's just more… getting clinicians to use it. (P8)
**Consultation support: Decision support for use with patients**
**Shared decision making:** I think shared decision making does [patient education] okay. … it's not easy to do, but I think that's the gold standard approach. And if the patient decides after you've gone through a careful shared decision that for them in their situation that day, they're prepared to take the downsides of antibiotics because of the small chance of a benefit from them, then all well and good, you let them have the antibiotics. You're not creating a platform of conflict there but next time the situation might be different, and there may not be the wedding to go to at the weekend or the sort of discussions that happen, and hopefully the patient will and GP will repeat the same conversation and conclude not to use them or to or to delay the use of them. So it's a chipping away continuous process of changing hearts and minds which can be done without conflict. (P7)
**Well-resourced shared decision making**. I think the decision support for practitioners and for patients is probably a key thing that we need to improve more because I think there's still that message that sometimes the quickest way to end the consultation is just give them antibiotics. Whereas now you can do that shared decision making. If it's been well thought through and they're well-resourced you can do it just as quickly potentially as giving the script for antibiotics. (P2)
**Evidence-based symptom management tools at hand**: Having a symptomatic management sort of prescription as an alternative to receiving antibiotic prescriptions, [a] patient action plan for managing their upper respiratory tract infection... I guess having the right information at hand as well to convey to the consumer at the time as well about why antibiotics are likely to be more harmful than good. (P5)
**GP-Patient technology**: A doctor has an app and the patient has an app and you just click a few things and then it goes straight to the patient's phone and they have… reminders for medications and… reminders for appointments… results… offer [to] send them videos and electronic information. (P6)
**Use the resources we have**: I think you've got to remember … the consultation is not particularly long. So you don't want to be making it something that takes your consultation 25 minutes… I just think we've got some really good resources out there. So, we're probably a bit lazy using them…. the NPS things are approved. They've been tested with consumers. They're a trusted brand. (P8)
**Potential developers of patient information**: The combination of people that are writing specific guidelines should be writing patient information packages to go with those guidelines. So I think when the College of GPs writes a guideline like the Red Book [*Guidelines for preventive activities in general practice*] it should have patient facing components that GPs can use as part of their toolkit and as part of the implementation strategy. So then I think a deal done with the Therapeutic Guidelines people for example would lead into particular patient facing pieces so that patients can check from a responsible source what the same information that the GP should be referring to. And I think big organisations that are doing this quite well like Health Direct could be employed to do more of it. The Victorian Better Health Channel and so on. So I think there are a number of organisations that that might be suitably unbiased and used to producing patient resources and testing those. The National Health Service have a number used in the UK for example. The National Prescribing Service is actually funded to do this sort of work so they could be producing patient information leaflets. Anything like that should be tested in you know rapid easy comparative trials rather than just brought out because it seemed like a good idea at the time. (P7)
**Where should patient information sheets be hosted?** Everywhere was the answer to that. So that… people access for them health information and optimized for Google searching and linked to the decision support. And I think if antimicrobial stewardship was seen as an important thing to promote, then even community pharmacists; although I think you need to be very careful that they're not doing that for commercial reasons to sell probiotics and complementary alternative medicines with little approval value. (P7)

**Hosting information in PHN Health Pathways:** Health Pathways is a New Zealand product which is then localized for use in Australia... it's really that one stop shop that has clinical guidance, it has access to consumer resources, and it has access to referral pathways, so really the GP can access everything through that but at this stage it's not really up to scratch for antibiotic use. (P4)
**Who should keep patient information updated?** Need a central clearing house… of accepted and reliable ones that are up to date. And organizations need to have a expiry date on them. But I don't like the idea of creating policy off the top of my head. I think it's best created by groups of people thinking about all the pros and cons. (P7)
**Culturally and linguistically appropriate patient information**: I think one of the problems with the health information on leaflets and things like that is that it is one size fits all. And if you really want to get through to people, one size fits all is not the approach. And especially if you're dealing with people from diverse cultural and linguistic backgrounds. So just translating something from English into their language… is not enough. Even in the Australian population one size doesn't fit all. We need all kinds of different approaches. (P9)
**Consultation support: Delayed prescribing and watch-and-wait strategies**
**Need for watch and wait**: GPs need education about the use of watch-and-wait. (P12)
**Better evidence needed for delayed prescribing**: What are the pros and cons for your patients as an individual, on having antibiotics? What's the natural course of the disease they've got without? What are the effective safety netting discussions to have?... So the message that it [a delayed prescription] might send if it's not communicated really well is you've not being sick enough for long enough to earn your right for an antibiotic. Please be sick for three more days and then come and have an antibiotic… I think delayed prescribing would only really work if the scripts are dated and date stamped with a limit on them rather than being open ended for up to a year. (P7)
**Pharmacist involvement**: Delayed antibiotic prescribing, so if that's going to be successfully implemented, it needs to have that communication process so the pharmacist is aware when they get that prescription it's not to be filled immediately and it should be a bit of counselling around it. But how are they… to be informed about that and what sort of communication strategies can the practice set up with the pharmacy? (P10)
**Consultation support: Pathology testing and reporting including rapid tests**
**Indication for tests**: Just because you've got a test available if without the test you would never have gone near antibiotics. You wouldn't want to have that decision altered by the test and also you may find that many of the bacterial infections are just as self-limiting as viral (P7)
**Need for support**: studies that showed that just putting [point of care tests] in the practices doesn't actually work without a whole lot of guidance and support around them. So yeah, I feel like there's a lot of people who think that those kinds of technological answers are the be all and end all to this; and I would thoroughly disagree. (P6)
**Availability and cost of rapid tests**: For a GP, rapid tests are not as rapid as if the patient has presented to ED… They are too expensive to do in general practice. (P12)
**Rapid tests with watch-and-wait**: If the GP feels it is OK to wait before prescribing antibiotics and watchful waiting is done, the GP can then review rapid tests to guide future treatment. (P12)
**Rapid diagnosis and communication of results**: I certainly don't think that rapid diagnosis is the be all and end all. But… a swab that said you have got human metapneumovirus or you have got respiratory syncytial virus or whatever… here… is the diagnosis, you have got this... therefore, antibiotics are not going to help you. So, I think rapid cheap diagnostics would help a lot. And I think mechanisms to communicate those diagnostic results that don't necessarily involve a patient tracking back to the GP having another appointment… you know in particularly in areas where GP don't bulk bill return visits. Those are all barriers. (P6)
**Responsibility**: Microbiology testing and reporting often means a combination of the different colleges communicating with each other is often how that improves (P3)
**Resource developer**: The [Royal] College [of Pathologists of Australasia could] do… e.g. a document for laboratories to provide to patients, comments to guide prescribing, diagnostic stewardship – when not to send specimens to the lab (P12)
**Consultation support: Allergy testing**
**No large impact on AMR**: I think it's important and I think there needs to be structures in place for it to happen, but it's one of those one patient at a time things and from my public health mind I don't feel like that will have a big an impact as you know hundreds and thousands of people (P6)
**Consultation support: Expert advice**

**Dependent on relationships**: Expert advice for me is very dependent on relationships that I built when I was in the hospital system. So if you've got a good network of experts you can call on but you know from an infection perspective it's… reliant on the goodness of… them giving you their time... (P6)
**Patient referral, advice line**: I've had patients that I've referred to infectious diseases who are waiting months. Now that's a sticking point… whether or not the government would be interested in having access lines for antibiotic resistance… if someone could ring them up … and get advice probably wouldn't be a bad thing. (P8)
**Standard information for GPs**: [Private pathology] had one doctor rostered on who answered calls all day. Registrars in [hospital] lab do answer GP calls, but… need something to guide GPs to direct them when they call. We need one official form rather than lots of different ones – they are not as strong as one consistent message. (P12)
**Pharmacists: Dispensing antibiotics**
**Information & data**: [pharmacists] don't know why patients are taking the antibiotics... if people should actually be recording... very distinct instructions on the script, then you're going to be able to capture that data in your data mining and then it will also go on to better use of antibiotics at the pharmacy. (P8)
**Unit dispensing**: Unit dispensing sits under a couple of different things some… sort of legislative basis and some that have a basis in the pharmacy agreement… However, the other way is there's always that back way, of if doctors write on their script three tablets. Then only three tablets get dispensed so that's where there's potentially a regulatory response or there's a cultural response of how people change their prescribing… But that's one of the ones where there's actually two ways of reaching the same endpoint. (P3)
**Unit dispensing: breaking packs**: I guess the issue is that they then got to suppose another patient will come in and get free and if another patient doesn't come in and get free then they've got to put it in the bin. (P8)
**Pack sizes**: We don't have a factory to make that size [pack] (P8)
**Pharmacist check with GP**: a script that says… take for five days one BD [twice a day] and then the GP has actually written the script for 20 keflex because that's how the script pack is. So for the pharmacist to then say look you're going to get a spare 10 please don't take them. That's a conversation to be had at the pharmacy level or say look you've got a spare 10, do you want me to ring your GP and see if he only wants 10 for size. You know that is so important and that doesn't happen. (P8)
**Pharmacist: Appropriate disposal of leftover antibiotics**
**Unaware**: I don't think a lot of people are aware that you can return unused antibiotics to a pharmacy. That's obviously something that you could do quite simply and… it doesn't require any changing of legislation or a lot of money to do something like that. (P4)
**Incentive**: trying to create a public will to return your leftover antibiotics from both veterinary practice and from human medicine. Even getting some money back on the dispensing fee might be a very modest and cheap way to reduce the number of antibiotics sitting around in people's nursing homes in in people's homes. (P7)
**Pharmacy review & advice to patients**
**Triage**: [Pharmacists] triage people… we are accessible most days of the week if not all days of the week. And it's easy to just pop into a pharmacy. And we do have a lot of training in our university course for it. (P1)
**Safety measure**: I think they [pharmacists] can do more around medication management and qualities of medicines… there's issues around having somewhere practical to have sensitive discussions... for things like urinary tract infections, around STI infections… pharmacists have good knowledge about medicines, but their… skills at diagnosis - they're not doctors… The fact that you have … a GP prescribing and then a pharmacist dispensing provides that extra safety measure, that you do have an extra person checking the appropriateness as well. (P4)
**Not asking patients for information**: [GPs and infectious disease physicians don't] want the pharmacists asking the patients [about their prescriptions] and then making their own judgements because they don't have that whole information… they don't know about other comorbidities… which is not their fault, because that's not their role… It shouldn't be [the patient's] job to try and remember why it was prescribed… If [GPs] put that reason on [the prescription] as you put… duration… it would help with the pharmacy dispensing for pharmacists to do the counselling. (P8)
**Pharmacists working with GPs**

**Funding for pharmacists in general practice:** There is a new workforce incentive… payment that's coming in in January [2020] which will allow pharmacists to [be employed by a general practice]. But it's coming from the same bucket of money as payments for nurses, so it's not really anticipated there's going to be a big uptake at this stage. The nurse role is more developed in general practice and additionally the nurses are revenue raising whereas the pharmacists generally aren't. (P4)
**Pharmacists in general practice**: Pharmacists working in general practice. They'd have a role both educating patients but also the GPs and other practice staff; but also the potential is for them to be involved in auditing and quality improvement activities around antimicrobial prescribing. (P4)
**Relationship**: it also that relational building between doctors and pharmacists, cos where there's no relationship it's very difficult for pharmacists to ring up a GP and say I think you made the wrong decision. Whereas if they've got a good relationship already there, then that might happen or if they've got a relationship with the practice they might have a regular route for feedback, saying look I've noticed that a lot more fluroquinolones coming through recently, what's been going on here guys? Why have you changed your practice? But in big cities… often, it can be difficult. So I think that is that is a cultural change at doctors being willing to hear from pharmacists but also pharmacists having the time and willingness to bring it up. (P3)
**Tension**: … you might get a pharmacist calling … and questioning [a GP's] prescription, who at the same time is peddling completely non-evidence-based supplements and all sorts of rubbish to… patients and charging them an arm and a leg to do so. (P6)
**Supply and timely access to antibiotics**
**Antibiotic out of stock**: There's also been in this past 18 months some antibiotics not being available so [pharmacist] ringing the doctor getting an alternative because the usual ones are out of stock. (P1)
**Nurse triage**
**Community based nurse:** I mean they're not going to the nurse [in the pharmacy] to get antibiotics but I suppose it's more perhaps for that reassurance that they don't need to go to emergency, and they don't need antibiotics. (P4)
**Monitoring of Antibiotic Prescriptions**
**Changes behaviour**: You look at the literature, now audit and feedback is a really good methodology for changing behaviour. (P11)
**Complete datasets**: One of the areas where I think actually government has a role in terms of getting complete datasets. Really I can't see it happening without at least a degree of regulation. (P6)
**Private prescriptions**: The bit about [monitoring] prescriptions can happen, either the practice level, or at the PBS level but that doesn't capture then private prescription… I don't think the PBS prescribing at the individual level is terribly useful at all. (P3)
**Use MedicineInsight**: I think the MedicineInsight program is a good one, there's still some issues to iron out … but …. that also has to do with the way software packages are set up and how we enter data into our medical records. But, it has taken a number of years to get to where they are now. And I don't think we need to invest in others to do exactly the same. I think we need to look at you know is this tool useful enough or developed enough to roll out on a much larger scale. (P9)
**Against NPS MedicineInsight**: No I don't [think NPS should do the monitoring]. I think that they're one of many. (P7)
**Reason-for-prescription**: some of the software really isn't designed to make it easy to fill [reason for prescription] in either. … sometimes … you've got to think up an obscure name for that particular condition for it to accept it. Things like that don't encourage reporting of reason for prescription. (P3)
**Software improvements**: once [the reason-for-prescription] gets written into the software then the extraction tools start to become more useful and then you have the ability to be able to do the feedback and reporting. (P3)
**Funding**: So, I think that [monitoring is] one of those things which needs a big chunk of investment so that we can get it off the ground effectively. (P6)
**Patient consent**: how much consent do we need to get from patients? (P2)
**GPs defensive**: I think GPs are… very protective of their data from a patient's point of view because there has been times when patient information has been misused… So I think there has to be done fairly sensitively… there is no suggestion it could be misused but… GPs... get a bit defensive and feel like they've been shamed… if we can separate it somewhat from

antibiotics

government or you know regulation people then… GPs will get less defensive about it…. But logically I guess it's going to be only certain people, isn't it… So, it's probably going to have to involve government in some ways (P2)
**Low volume prescribers**: if you're looking at high prescribers you can look at low prescribers as well you know (P9)
**PIP QI incentive:** Maybe [monitoring] needs to become part of the QI PIP so that there's a carrot to do it. (P8)
**SafeScript model**: The SafeScript model that's being implemented for opiates and other drugs of addiction is something that could be considered as a component in general practice. It's probably a little bit more hardcore but a modified version of it could be a way of both collecting data and restricting prescriptions. (P6)
**AMR**
**Laboratory monitoring of AMR, use of PCR**: Monitoring of resistance tends to be best done capturing it through the labs… that gets analysed at the state level… and at AURA which is at the Commission [ACSQHC]… I think that some of the [gaps are] the real time monitoring. Yeah and there's definitely a gap in general practice resistance that goes to private pathology labs… There's a few gaps that are developing due to the use of PCR rather than culture and resistance testing. (P3)
**Complete datasets**: There needs to be an overarching Government responsibility from a regulatory perspective… to get complete datasets... There's lots of passive surveillance systems that get a pretty good view of things but not a complete view of things. You know the organisms for which we really have absolute datasets for are the things that are notifiable… And from one health perspective we need to be able to integrate and it's becoming more and more important that we can integrate surveillance of isolates and resistance across human and animal and food and environmental and effluent and all of those… different sources… the prevalence of resistance is a is a key one that we need that we want to know about. (P6)
**Data feedback to GPs**
**Patient outcomes**: PBS… MedicineInsight… doesn't give us follow up data.. You can tell doctors… your colleague has prescribed less than you, then they'll say yeah, but are the… patient outcomes just as good or better or worse than mine? And we can't provide that answer. (P9)
**Relevant comparisons**: we need to be mindful that people working in [e.g. Aboriginal Health] that if we compare their antibiotic prescribing to their colleagues in you know urban environments that's not the right thing to do…. I really think that comparison to peers works really effectively. And also just showing people you know where they can improve upon, and also I suppose where PHNs can target. Without the data it's hard to tell who you need to work with. (P9)
**Link with education & support**: [Data extraction] will need to be combined with a cultural change - another one - which links back into the education and the consultation support about adding reason for prescriptions in… (P3)
**Feedback on positive variance**: But there's nothing that tells us if we are you know being a good custodian… if we're doing the right thing we don't know... I think that that is important. (P9)
**Self-monitoring**: I think every doctor should be looking at their own data on a regular basis, reflecting and learning from that... I would make antibiotics a mandatory part. You know everyone has their audit on may be on a yearly or maybe on a triennial basis, audit their prescribing for antibiotics and… use that as a way to improve the quality of the care they provide. (P9)
**Responsibility**: I think more regular feedback and yeah, I think PHNs are well placed to do that with the... [PIP QI] that practices are reporting to PHNs; and they will be getting quarterly reports comparing them against similar practices. (P4)
**Embedded pharmacist feedback**: From a quality of prescribing point of view, at the entity level of the general practice it should be a pharmacist embedded within that environment whose responsibility to do [feedback]. (P11)
**Research**
**Hospital admissions:** Unintended consequences such as hospital admissions for things that could have been prevented with antibiotics. (P3)
**Patient knowledge**: I wonder if people who've been doing it for longer and you know much more experienced, perhaps forget that patients don't necessarily know these basics… I suspect we assume knowledge that is not necessarily there. (P6)
**Gut microbiome; Self-limiting infections**: the biome of the gut… could be useful to direct and encourage people to prescribe less antibiotics but also understanding potentially the negative effects of antibiotics… I think the other research thing is… about the self-limiting nature of [disease]. (P2)

**Research translation & Patient information resources**: There's a keen interest obviously to translate research to something that can be picked up… we need to work on [patient information sheets] continuously in terms of research context and then… how do we implement them nationally when they reach that level of value and evidence behind them? (P5)
**Biggest impact**: It is complicated, what will have the biggest impact? I'm not sure, research is needed… Some areas are more powerful than others – whatever the research focus says is the most important and some, e.g. disposal of antibiotics, will have less of an impact. (P12)
**Real time monitoring and Effect on AMR**: we need a much better way of understanding for which patients and why things are being prescribed and that's why an in-depth NPS MedicineWise type system that works in real time and is less clunky, would be useful to assist primary care. The magic question is can we reduce the development, spread of antibiotic resistance through a reduction in antibiotic prescribing? And then there's a question of whether we should be rotating antibiotics globally and say right these antibiotics are going to be put away and not used until resistance has dropped. And then we'll take them out and put some others away in a cupboard. But you'd have to understand the rate of extinction of antimicrobial resistance. (P7)
**Pilots**: I think there does need to be more of the small-scale piloting things so that we can ensure that what is planned is working before it is rolled out more broadly. (P4)
**Evaluation**: research to see whether or not campaigns have an impact... (P8)
**Measures of success**
**AMR rates**: There's three levels of outcomes. The least ambitious one would be that we… slow down the development of resistance and the spread of resistance and that the accelerating decreases. The second tier is somewhat more ambitious, is that we stop the development of resistance and it just stays where it is now. And the third level is that we that we reverse it. All of which depend on having actually decent amounts of surveillance information data so that we've got an actual idea of where we sit at the moment. So probably the first the first thing is that we would see an increase because if we collect the data properly we will have a better idea of what the levels of resistance are. But once we've got an accurate picture in the ideal world, we would reverse resistance. In a more realistic world if we could… slow the acceleration that'd be nice. If we can help it and keep it at current levels that'd be better. (P6)
**Decreased AMR and prescribing; patient outcomes**: Are we prescribing less, how are we going with our resistance? We'd have to of course also look at your overall outcomes in patients. (P9)
**Decreased AMR; Appropriate antibiotics**: Well I would hope that antimicrobial resistance would slow, and antibiotics would be used more appropriately but that would be the first stage. But if you saw antimicrobials would be prescribed and used more appropriately and hopefully that would slow the decline of resistance. (P4)
**Empowered GPs decrease prescribing**: GPs would be empowered to decrease antibiotic prescribing and that should reduce antimicrobial resistance. (P12)
**Professional satisfaction:** If you want to make people accountable, responsible, then your outcome should also be looking at levels at the level of the prescribers, and their satisfaction is maybe not the right word, but their support and their confidence, and in how it has impacted their relationship with their patients. So maybe that's the one thing that we haven't really looked at, we have those big data stuff which is very important but success is also depends on how the people who enable it or enact it how they feel about. (P9)
**Hospitalisations**: I think the way Strama has done it is really nice, the way they also looked at some collateral damage. And the UK have done it to a certain extent. It's probably easier because they have a more centralized approach so it's easier to see who gets admitted to hospitals or who comes back to GP practices if they haven't received an antibiotic for their condition. I'm not too sure how we could track that in Australia. (P10)
**Patient outcomes**: I think there'd be plenty of really good positive things for the patient really. You know like consultation support stuff I think would make a really big difference… (P2)
**Multiple measures: I**f one of your aims was changing knowledge, you test knowledge. If you want a changed attitude, you look for changed attitudes. If you want to test add[ing] skills to prescribers and dispensers, you look for those skills. If you want to look at your surveillance system, you look how effective the surveillance system is. And if you want to look at unintended consequences, you look at hospital admissions and deaths from infection and you look at cases of overwhelming infection and see whether it was a missed opportunity to prescribe antibiotics at an earlier stage. I think if you want to look for the final common pathway… you can look at the patterns of resistance and see if you can actually demonstrate some improvements in resistance patterns through efforts and whether you can track those to where the processes are happening most. (P7)
